# GC-MS Study of the Chemical Components of Different *Aquilaria sinensis* (Lour.) Gilgorgans and Agarwood from Different Asian Countries

**DOI:** 10.3390/molecules23092168

**Published:** 2018-08-28

**Authors:** Meng-Ru Wang, Wei Li, Sha Luo, Xin Zhao, Chun-Hui Ma, Shou-Xin Liu

**Affiliations:** 1 Key Laboratory of Bio-Based Material Science and Technology (Ministry of Education), College of Material Science and Engineering, Northeast Forestry University, Harbin 150040, China; nefuwangmengru@163.com (M.-R.W.); liwei19820927@126.com (W.L.); luo.sha.85@163.com (S.L.); 2 Key Laboratory of Pulp and Paper Science & Technology of Ministry of Education/Shandong Province, Qilu University of Technology, Jinan 250353, China; zhaoxin_zixi@126.com

**Keywords:** *Aquilaria sinensis* (Lour.) Gilg, volatile component, alcohol extracts, GC-MS, antioxidant capacity, antibacterial activity

## Abstract

As a traditional medicinal herb and valuable natural spice in China, *Aquilaria sinensis* (Lour.) Gilg has many significant pharmacological effects. Agarwood is the resinous heartwood acquired from wounded *A. sinensis* trees, and is widely used in pharmaceuticals owing to its excellent medicinal value. In this study, the chemical composition of volatile components and alcohol extracts from different organs of *A. sinensis* and agarwoods grown in different regions were investigated using GC-MS. The results showed that Vietnam agarwood had the highest moisture content, which was attributed to the local climate, while the fruit and bark of *A. sinensis* had higher moisture contents than the other organs. The volatile components of *A. sinensis* organs included 3-ethyl-5-(2-ethylbutyl)-octadecane, oleic acid 3-(octadecyloxy) propyl ester, and docosanoic acid 1,2,3-propanetriyl ester, while the alcohol extracts of *A. sinensis* organs contained benzoic acid ethyl ester, hexadecanoic acid ethyl ester, oleic acid, and *n*-hexadecanoic acid. Furthermore, the main active ingredients in agarwood from different habitats were sesquiterpenoids, aromatic species, and chromone compounds. The role of chromone compound 2-phenylethyl-benzopyran as an elicitor and the mechanism of agarwood formation were also investigated. Antioxidant tests showed that essential oils from agarwood and *A. sinensis* had antioxidant capacities by comparison with butylated hydroxytoluene and vitamin E. An antibacterial activity test showed that the inhibition effect of the essential oil was better against Gram-positive bacteria than against Gram-negative bacteria.

## 1. Introduction

*Aquilaria sinensis* (Lour.) Gilg (*A. sinensis*) is a tropical evergreen tree native to China that has been widely used as traditional medicine in East and Southeast Asia for hundreds of years. The resinous portion of *A. sinensis* branches and trunks, known as agarwood, is widely used in traditional medicine as a digestive, sedative, and antiemetic, and is also used in incense and perfume [1,2]. Agarwood is a resinous material collected from *A. sinensis*. In wild forests, only 7–10% of *A. sinensis* trees can produce agarwood, and healthy *A. sinensis* cannot produce agarwood. Agarwood is secreted as a black resin with an aromatic fragrance only when *A. sinensis* is stimulated by external stimuli, such as physical or chemical damage or endophytic bacteria [3,4]. A drastic decline in the number of *Aquilaria* trees in natural forests has earned it endangered status. Agarwood resin is widely used in medicine, cosmetics, food, and other fields [5]. As a famous traditional Chinese medicine and a highly valuable non-timber product, the demand for agarwood is much greater than its supply [6]. At the end of last century, *A. sinensis* was planted for artificial cultivation in Hainan, Guangdong, and in other regions [7,8,9]. *A. sinensis* has been shown to contain chemical components such as terpenoids, flavonoids, lignans, and steroids [10,11]. Furthermore, an impressive body of literature indicates that the main components of healthy *A. sinensis* are fatty alkanes, while the main components of agarwood are sesquiterpenoids and aromatic species [11,12,13,14,15,16]. Chemical investigations into agarwood have shown that sesquiterpenes, 2-(2-phenylethyl)-4-*H*-chromen-4-one derivatives, and aromatic compounds are the main characteristic chemical constituents [7,17,18,19]. 

Existing literature on agarwood is mainly focused on studying the types and structures of two pharmacologically active components, namely, 2-(2-phenylethyl)chromone derivatives and sesquiterpenes [15,17,20,21]. However, few studies have compared the contents of these two compounds in agarwood from different regions. Furthermore, research on *A. sinensis* has mainly focused on leaf and seed chemical composition [1,22], while the chemical composition and volatile contents of different organs of *A. sinensis* have not been systematically studied. Therefore, herein, the chemical composition of volatile components and alcohol extracts of different organs of *A. sinensis* and agarwood from different regions were investigated by GC-MS. Furthermore, the antioxidant and antibacterial activities of their essential oils were tested. This study aimed to develop a method for identifying the chemical composition of agarwood, providing a theoretical basis for further experiments, and to compare exotic agarwood species and understand the main chemical components in different samples. The results will provide a basis for selecting the best quality agarwood from different origins and a foundation for the quality evaluation and mechanism study of agarwood.

## 2. Results and Discussion

### 2.1. Moisture Analysis of A. sinensis Organs and Agarwood from Different Regions

Three precision-weighed (1.00 g) powdered samples of agarwood xylem from different regions and different *A. sinensis* organs (including blossom, seeds, peel, leaf, branch, xylem, bark, and root) were dried to constant weight in an oven at 105 ± 3 °C to calculate their average moisture contents. 

Agarwood can resist external attack of the xylem parts and its moisture content is lower than that of xylem. The main factors influencing the moisture content are ambient humidity and the agarwood formation mechanism. Compared with other regions, the moisture content of Chinese *A. sinensis* agarwood was the lowest, at 7.99 ± 0.13% (Table 1). *A. sinensis* agarwood is mostly grown in areas near the Tropic of Cancer, which has a humid tropical and subtropical monsoon climate with high temperatures and abundant rainfall. *A. sinensis* agarwood is also commonly grown in mountain rainforest or semi-evergreen rainforest with moist, porous, and humus thick soil. Agarwood from Vietnam had the highest moisture content (14.73 ± 0.08%). This was attributed to Vietnam having a tropical monsoon and tropical rainforest climate, with four distinct seasons in the north, while the four seasons are divided into dry and rainy seasons in the south owing to the effects of the monsoon. Furthermore, the moisture contents of Malaysian and Indonesian agarwood were 9.22 ± 0.16% and 11.27 ± 0.31%, respectively. Malaysia is located near the equator and has a tropical rainforest climate and tropical monsoon climate without obvious seasons, with average temperatures of 26–30 °C and abundant rainfall. The Indonesian climate is similar to that of Malaysia and typical of a tropical rainforest climate with abundant rainfall. These fundamental climate factors account for the different moisture contents of agarwood from different regions. Furthermore, fresh samples recently obtained from agarwood had a relatively high moisture content. As the weight is not the same as the density, it is easy to submerge at the place of production. Once separated from the tree, the water supply is cut off. However, owing to the influence of outside temperature, the moisture stored in the interior is gradually reduced. Agarwood of good provenance is not likely to sink. The moisture content of agarwood has a significant influence on its weight, which results in the weight of agarwood changing during rainy and dry seasons. The original water content of agarwood is relatively abundant, and its weight can be reduced under dry conditions and during storage. This is an important reason for selecting agarwood is the lower moisture content, and the higher quality [23]. Furthermore, testing the different organs of *A. sinensis* showed that the root had the lowest moisture content (46.48 ± 0.36%). This was attributed to the root comprising the water transport channels of the plant, meaning that it does not retain water. The fruit had the highest moisture content, including the blossom, seed, and peel. Owing to the driving force of transpiration [19], the moisture contents of the blossom, seed, and peel were 71.98 ± 0.83%, 79.72 ± 1.27%, and 81.20 ± 0.68%, respectively. The moisture content of the bark was 70.07 ± 0.23%, while those of the leaves and branches were 63.88 ± 0.66% and 63.23 ± 0.24%, respectively. The moisture content of the xylem was lower, at 51.95 ± 0.03%.

### 2.2. Chemical Composition Analysis of A. sinensis

**Figure 1:**
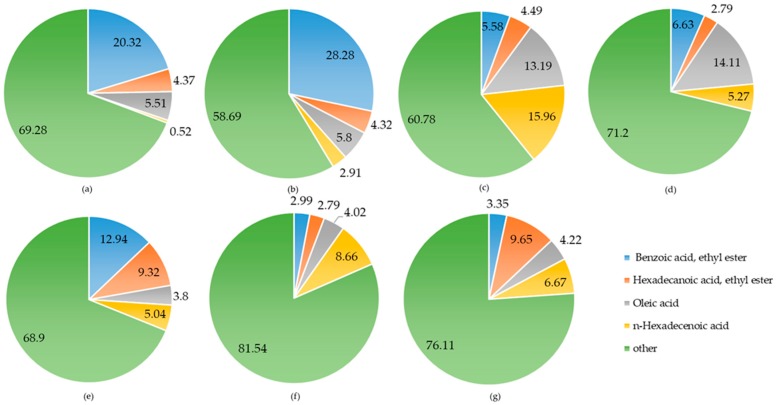
Chemical composition of alcohol extracts of different *A. sinensis* organs: (**a**) root; (**b**) peel; (**c**) blossom; (**d**) bark; (**e**) branch; (**f**) blade; and (**g**) seed.

**Figure 2:**
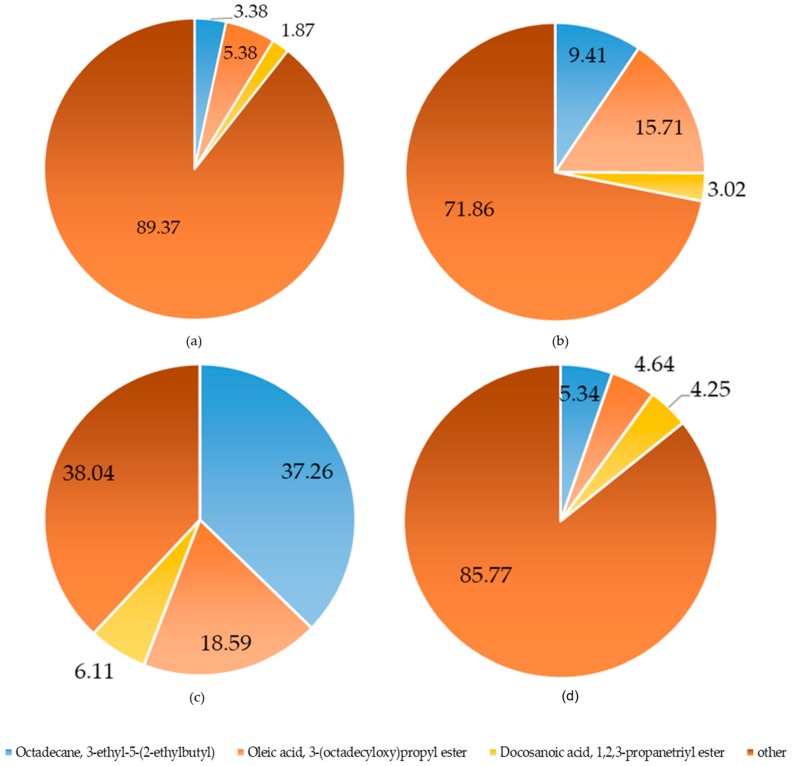
Chemical composition of volatile components in different *A. sinensis* organs: (**a**) peel; (**b**) blossom; (**c**) blade; and (**d**) seed.

Samples of *A. sinensis* organs, prepared according to Section 3.2.2, were tested by GC-MS to determine the yields of volatile components and alcohol extraction. The main composition of the alcohol extracts and volatile components of *A. sinensis* organs are shown in Figure 1 and Figure 2. The volatile components and relative percentages were measured and are listed in Table 2.

The alcohol extracts of different *A. sinensis* organs (Figure 1) contained benzoic acid ethyl ester, hexadecanoic acid ethyl ester, oleic acid, and *n*-hexadecanoic acid. Among them, benzoic acid was abundant in the root (20.32%) and peel (28.28%). Compared with the literature [24,25], the RA(%) value of Benzoic acid, ethyl ester was 14%, while the data in Table 2 is higher than the literature data, whereas the hexadecanoic acid ethyl ester contents in the branch and seed were similar, at 9.32% and 9.65%, respectively. As shown in Figure 1, oleic acid was concentrated in the bark (14.11%) and *n*-hexadecanoic acid was concentrated in the blossom (15.96%). Because the RA(%) values in other organs of *A. sinensis* were not listed in the previous studies, only the chemical structures have been analyzed, so no comparison could be made. Inorganic compounds are transported upward in the xylem of plants, while organic matter is transported through the phloem upward and downward, with preferential transport to the growth centers of the plants [26]. Owing to plant transpiration, water is transported from the bottom to the top. The organic acid contents are higher in the leaves and blossom because organic acids are water soluble, while the water solubility of ester organic compounds is poor, resulting in higher ester contents in the roots and leaves [25].

The main volatile components in the different organs were 3-ethyl-5-(2-ethylbutyl)-octadecane, oleic acid 3-(octadecyloxy) propyl ester, and docosanoic acid 1,2,3-propanetriyl ester (Figure 2). These three compounds all have obvious antimicrobial effects and potential bioactivities [27,28]. Therefore, vulnerable external parts of the plant body are rich in these compounds. The 3-ethyl-5-(2-ethylbutyl)-octadecane content was highest in the blades (37.26%), while its contents in other organs were in the order: blossom (9.41%) > seed (5.34%) > peel (3.38%). The oleic acid 3-(octadecyloxy) propyl ester content was highest in the blade (18.59%), while its contents in the blossom, peel, and seed were 15.71%, 5.38%, and 4.64%, respectively. Furthermore, the docosanoic acid 1,2,3-propanetriyl ester content was highest in the blade (6.11%). 

The chemical composition and relative percentages of the contents of *A. sinensis* alcohol extracts, prepared according to Section 3.2.3, were determined by GC-MS and the results are listed in Table 2. The results show that the volatile components of the blossom (16.59%), seed (21.30%), peel (21.50%), and blade (13.34%) contained a high abundance of fatty acids, such as benzoic acid, and 10-octadecenoic acid methyl ester. Similarly, the alcohol extracts of the blossom (15.96%), seed (36.66%), peel (28.28%), and blade (8.66%) contained *n*-hexadecanoic acid and squalene. Squalene has strong oxygen capacity, antifatigue, and anticardiovascular disease effects [29]. The main chemical constituents in the blade were myristicin and palmitic acid. In a preliminary study by Wu and coworkers [13], *A. sinensis* peel extracts showed obvious antibacterial and antitumor activities. However, the proportion of these compounds in the branches, xylem, bark, and root was very low.

### 2.3. Chemical Composition Analysis of Agarwood from Different Regions

Agarwood samples from different geographic regions were prepared according to the procedures described in Section 3.2.2 and Section 3.2.3, and tested using GC-MS. The volatile components and relative percentages of effective constituents were identified and are listed in Table 3.

**Figure 3:**
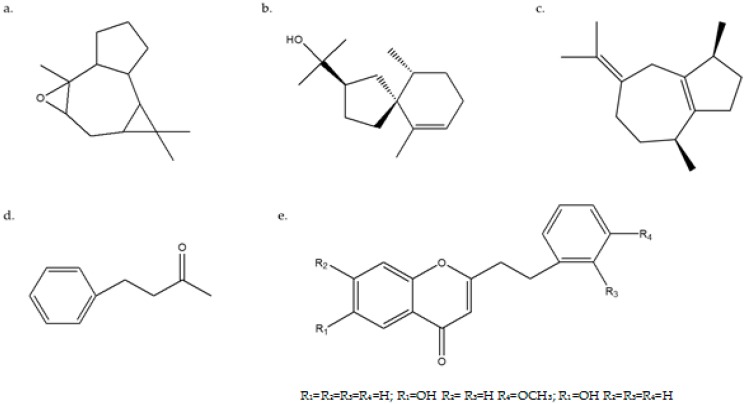
Structural formulas of compounds commonly found in alcohol extracts of agarwood from different regions. (**a**) isoaromadendrene epoxide, C_15_H_24_O; (**b**) agarospirol, C_15_H_26_O; (**c**) β-guaiene, C_15_H_24_; (**d**) benxylacatone, C_10_H_12_O; (**e**) 2-(2-phenylethyl)chromone, C_17_H_14_O_2_).

The chemical composition of agarwood has been studied extensively [30,31,32]. The chemical composition of agarwood from China was the same as that of imported agarwood. Analyzing the alcohol extracts of agarwood from different regions showed that they contained volatile oils, sesquiterpenoids, 2-(2-phenylethyl)chromone, fatty acids, and other components (Figure 3). Among them, the main volatile oil components in agarwood are sesquiterpenoids and aromatic compounds. 2-(2-Phenylethyl)chromone is a characteristic component that confirms subfamily Agaraceae as an independent subfamily, while its precursor diphenyl pentanone is widely found in plants of the Thymelaeaceae family [33].

The active ingredients for all agarwood obtained from different regions included sesquiterpenoids, aromatic species, and chromone compounds. For example, in Malaysian agarwood, chromone compounds accounted for 2.77% of the total content, while in Indonesian agarwood, chromone compounds and agarospirol accounted for 0.61% and 0.49% of the total content, respectively. In Vietnamese agarwood, the proportions of agarospirol and 2-(2-phenylethyl)chromone with its derivatives were 0.66% and 0.41%, respectively. The proportion of chromone compounds was the highest in Chinese agarwood (2.93%). 

**Figure 4:**
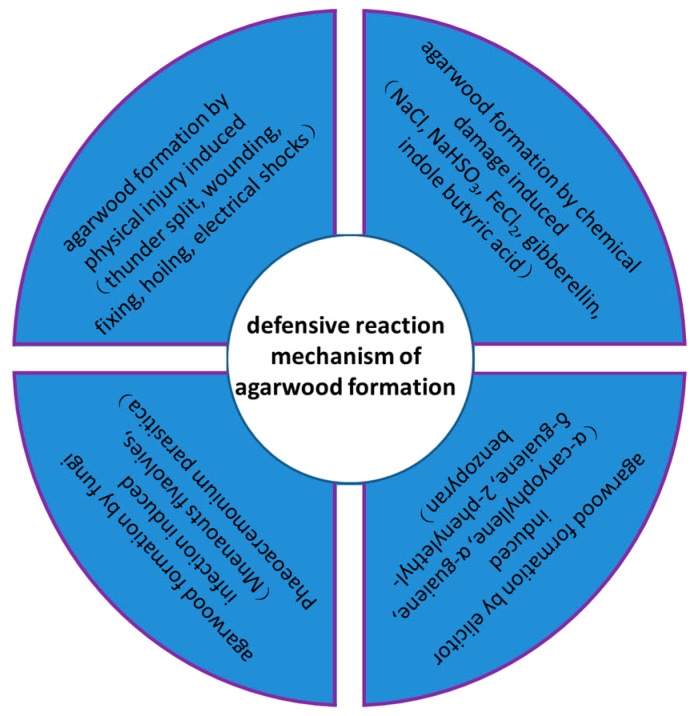
Defensive reaction mechanism of agarwood formation.

Sesquiterpenoids have been reported to have antineuroinflammatory properties, while aromatic species and chromone compounds have shown inhibitory activity toward human gastric cancer cells [34,35,36]. According to previous research, the defensive reaction mechanism of agarwood formation (Figure 4), can be induced by physical injury [37,38], chemical damage [39], fungi infection [40], or elicitors, as shown in Figure 4 [41,42]. Chromone compound 2-phenylethyl-benzopyran is the elicitor that induces agarwood formation. The different chromone compound contents among agarwood samples provides a scientific basis for the screening of Chinese agarwood.

### 2.4. Antioxidant Activity Tests

#### Free Radical Scavenging Activity Assay of the Essential Oil

**Figure 5:**
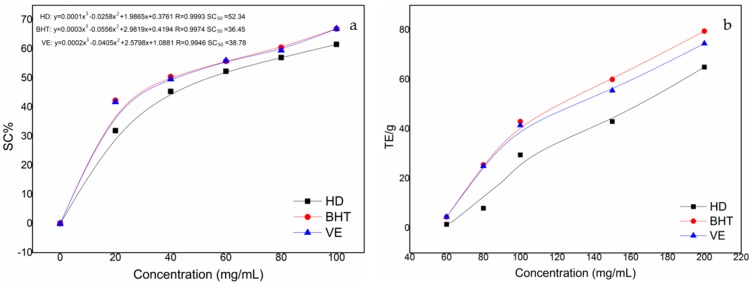
Results of (**a**) DPPH free radical scavenging activity and (**b**) ferric ion reducing antioxidant power (FRAP) assay.

DPPH is a highly stable nitrogen-centered free radical that can capture other free radicals, with a strong absorption centered at 517 nm. Freshly prepared DPPH solution is a deep purple color that becomes colorless or pale yellow after reacting with an antioxidant [43]. As shown in Figure 5a, with increasing essential oil concentration, the scavenging activity (SC%) increased significantly. When the concentration was less than 60 mg/mL, the scavenging activity increased rapidly, but as the concentration was increased further, the scavenging activity increased more slowly. The antioxidant capacity of the essential oil was much weaker than those of butylated hydroxytoluene (BHT) and vitamin E (VE). According to the literature, if a substance has an SC_50_ value (the concentration of essential oil when the SC% was 50%) of less than 10 mg/mL, the substance is considered to have an antioxidant capacity [44]. The DPPH scavenging effect decreased in the order: BHT > VE > HD (HD represents the volatile components extracted by hydrodistillation).

The antioxidant activity of the sample was determined using the ferric ion reducing antioxidant power (FRAP) assay established by Benzie and Strain [45,46]. The standard curve equation obtained by the test (y = 0.002x + 0.1049, *R*^2^ = 0.999) was used to determine the reducing ability of the extract. The reduction capacity of the sample was expressed as the FRAP value, with a higher FRAP value representing a better reduction capability. As shown in Figure 5b, the ferric reducing power increased with increasing concentration, and the antioxidant capacities were in the order: BHT > VE > HD.

### 2.5. Antibacterial Properties

In recent years, plant essential oils have received much attention in bacteriostatic compound research owing to their remarkable bacteriostatic activities and unique advantages of reduced side effects and residual toxicity compared with synthetic chemical bacteriostatic agents. In general, Gram-positive bacteria are more sensitive to plant essential oils than Gram-negative bacteria because the outside of the cell wall of Gram-negative bacteria contains a lipopolysaccharide layer that prevents hydrophobic compounds from entering the cells, which reduces the bacteriostatic effect [47]. As shown in Table 4, with increasing essential oil concentration, the *Bacillus subtilis* (*B. subtilis*) and *Staphylococcus aureus* (*S. aureus*) inhibition ratios became stronger, at 60.80 ± 3.82% and 64.46 ± 3.01%, respectively. Meanwhile, the inhibitory effect on *Escherichia coli* (*E.* *coli*) was weaker, at 57.97 ± 3.44%. The inhibition rates were in the following order: *S. aureus* > *B. subtilis* > *E.* *coli*.

## 3. Materials and Methods

### 3.1. Materials

#### 3.1.1. Apparatus

GC-MS analysis of the essential oils was conducted on an Agilent 6890N-5973 insert gas chromatograph (Agilent Technologies, Palo Alto, CA, USA) using an HP-5MS5% phenyl methyl siloxane capillary column (30 mm × 0.25 mm × 0.25 μm) and equipped with an Agilent 6890N-5973 mass selective detector in electron impact mode.

#### 3.1.2. Plant Material

Agarwood from Vietnam, Indonesia, and Malaysia was purchased from SanKeshu Medicinal Materials Market, Harbin, Heilongjiang Province, China, and authenticated using scanning electron microscopy (Quanta 200, FEI, Eindhoven, The Netherlands) by Yongzhi Cui, Northeast Forestry University. *A. sinensis* (diameter, 8.5 cm) was obtained from Guangdong. Different parts were separated and stored at −20 °C, and then underwent water distillation or Soxhlet extraction for 48 h after smashing. Climatic information about agarwood from different regions is shown in Table 5.

#### 3.1.3. Bacterial Strains

*E.coli*, *B. subtilis*, and *S. aureus* were obtained from the Food Microbiology Laboratory of Northeast Forestry University, Harbin, China. Bacterial strains were kept on an agar slant nutrient medium at 4 °C until use.

#### 3.1.4. Preparation of Culture Medium

A beef paste peptone culture medium was used in this experiment. This culture medium was prepared from beef paste (3.0 g), peptone (1.0 g), NaCl (0.5 g), agar (2.0 g), and water (1000 mL), and adjusted to pH 7.0–7.2 using 1 mol/L NaOH.

### 3.2. Methods

#### 3.2.1. Moisture Detection Method

Precisely weighed powdered samples of agarwood from different regions and different *A. sinensis* organs (1.00 g) were dried to a constant weight in an oven at 105 ± 3 °C to calculate the moisture content. Samples were measured in triplicate and average values were recorded.

#### 3.2.2. Hydrodistillation (HD) Method for Extracting Volatile Oils

Powdered samples of different *A. sinensis* organs (200.0 g) were added into a round-bottom flask with distilled water (1800 mL), and subjected to water distillation for 6 h using an electric heater (450 W). The essential oil was extracted with diethyl ether, dried with anhydrous sodium sulfate, and stored under cryopreservation conditions at −20 °C for further analysis.

#### 3.2.3. Soxhlet Extraction Method for Preparation of Alcohol Extracts

Powdered samples of different *A. sinensis* organs (10.00 g) were put in filter paper bag and subjected to ethanol Soxhlet extraction for 8 h. After evaporating the ethanol, the extracts were stored at −20 °C.

#### 3.2.4. GC-MS Analysis Condition

GC was performed using the following conditions: manual injection, 1 μL; injector temperature, 270 °C; carrier gas (He) flow rate, 1 mL/min; programmed oven temperature, 60 °C held for 5 min, ramped from 60 to 120 °C at a rate of 4 °C/min, held for 5 min, ramped from 120 to 170 °C at a rate of 3 °C/min, held for 2 min, and ramped from 170 to 280 °C at a rate of 10 °C/min. The detector temperature was 280 °C. MS was performed under the following conditions: Scan range, 15–500 amu; scan-TIC mode; injection port temperature, 250 °C; column oven temperature, 60 °C; ion source temperature, 230 °C; carrier gas (N_2_) flow rate, 1.6 mL/min; EI voltage, 70 eV; quadrupole rod temperature, 150 °C; quality scan range, *m*/*z* 40–400. The chemical compositions of essential oils were identified by direct comparison of their mass spectra in the NIST11 Mass Spectral Library.

#### 3.2.5. Antioxidant Capacity

##### DPPH Radical Scavenging Activity Assay of Volatile Oils (DPPH)

DPPH (3.5 mg) was made up to 100 mL with methanol in a brown 100-mL volumetric flask. Essential oil–methanol solution (1.0 mL) was mixed with DPPH–methanol solution (2.0 mL), and the mixture was shaken (100 rpm) and allowed to stand at room temperature for 30 min. The absorbance was measured at 517 nm using a TU-1900 UV spectrophotometer (Persee, Beijing, China) with methanol as the blank. The DPPH anion radical scavenging activity of the mixture was expressed as the SC%, which was calculated using the following equation:

(1) $$\mathrm{SC}\%=\frac{A_{0}-A}{A_{0}}\times 100\%$$

where *A*_0_ is the concentration of the antioxidant components in the *A. sinensis* essential oil extract before the reaction with free radicals, and *A* is the concentration of the antioxidant components in the *A. sinensis* essential oil extract after the reaction with free radicals. The oil concentration that provides 50% inhibition (SC_50_) was calculated from the plot of percentage inhibition of different oil concentrations. Experiments were performed in triplicate. BHT and VE were used as positive controls [48].

##### Ferric Ion Reducing Antioxidant Power (FRAP) of the Volatile Oil

Acetate buffer (300 mmol/L, pH 3.6), FeCl_3_·6H_2_O solution (20 mmol/L), and TPTZ (2,4,6-tripyridyl-*S*-triazine) solution (10 mmol/L) were mixed in a 10:1:1 (*v*/*v*/*v*) ratio to obtain the TPTZ working solution. Sample solution (0.3 mL) was added to TPTZ working solution (2.7 mL) preheated to 37 °C. The mixtures were shaken for 10 min and measured at 593 nm using a TU-1900 UV spectrophotometer (Persee). Before the experiment, the initial absorbance of the reagents (3 mL) and the acetate buffer (3 mL) were measured at 593 nm and used as blanks. Experiments were conducted in triplicate. The corresponding Trolox mass fraction obtained from the standard curve of the absorbance after the reaction is defined as the FRAP value (mg Trolox/g extract) [49].

#### 3.2.6. Antibacterial Activity

The bacteriostatic activity of the essential oils was tested by measuring bacteriostatic circles using the filter paper method. Qualitative filter paper was cut into small round pieces (diameter, 1.5 cm) using a puncher, and autoclaved before use. *E. coli*, *B. subtilis*, and *S. aureus* were cultured in nutrient agar medium at 37 °C for 24 h. Under sterile conditions, the corresponding culture medium (20–25 mL) was poured into the sterilized culture dish and the solid plate was prepared after cooling and solidifying. The prepared bacterial suspension (200 μL) was added to the corresponding solid plate medium and uniformly coated. The filter paper sheet was attached to the bacteria-bearing plate. Essential oil (5 μL) was added to each piece of filter paper. The essential oil diffused from the center to the periphery of the bacteria-bearing tablet, gradually forming a concentration gradient that inhibited the growth of the tested strains and formed a transparent bacteriostatic circle. The bacteriostatic activity of the essential oils can be preliminarily determined according to the OD of the bacteriostatic ring [50]. The inhibition ratios were calculated as follows:

(2) $$\mathrm{Inhibition}\;\mathrm{ratio}\;\left(\%\right)=\frac{R_{t}-Ro}{R_{t}}\times 100\%$$

where *R_t_* is the average OD of the inhibition zone, and *Ro* is the average OD of the blank sample. Experiments were performed in triplicate.

## 4. Conclusions

Agarwood is widely used in traditional Chinese medicine, incense, and perfumes. *A. sinensis* is the only certified source of agarwood products listed in the China Pharmacopoeia. The moisture content of agarwood from different regions, including China, Indonesia, Malaysia, and Vietnam, was investigated, with Chinese agarwood found to have the lowest moisture content and Vietnamese agarwood the highest. This result was attributed to Vietnam being located just south of the Tropic of Cancer, with a tropical monsoon climate and high humidity. Among the parts of *A. sinensis*, the moisture content of the root was the lowest, while that of the fruit was the highest. According to chemical composition analysis of *A. sinensis*, the seeds had the highest volatile component and alcohol extract contents. Squalene, fatty acids, and highly unsaturated fatty acids were abundant. The chemical constituents of agarwood from different regions were also analyzed. The results showed that all species contained active chemical compounds, such as sesquiterpenoids, aromatic species, and chromone compounds. The proportion of chromone compounds was highest in Chinese agarwood. Chromone compound 2-phenylethyl-benzopyran is a type of elicitor that induced agarwood formation. The antioxidant capacity of essential oils extracted by HD were determined and compared with traditional antioxidants. The DPPH scavenging effect decreased in the order BHT > VE > HD, while the FRAP test results decreased in the order BHT > VE > HD. In the antibacterial ability test of the essential oils, the essential oil concentration increased and inhibition rate decreased from *S. aureus* to *E.* *coli*. The present study identified the chemical compositions of agarwood and provides a basis for selecting the best quality agarwood from different origins. This study also provides a new approach to the identification of agarwood from domestic and exotic species.

## Figures and Tables

**Table 1:** Moisture analysis of *A. sinensis* organs and agarwood from different regions.

No.	Origin	Hydrodistillation Extraction (%)	Soxhlet Extraction (%)	Moisture Percentage (%)
1	China	ND	2.28 ± 0.16	7.99 ± 0.13
2	Malaysia	ND	21.47 ± 0.90	9.22 ± 0.16
3	Indonesia	ND	10.51 ± 0.36	11.27 ± 0.31
4	Vietnam	ND	11.5 ± 0.45	14.73 ± 0.08
5 *	blossom	<0.05	21.51 ± 1.05	71.98 ± 0.83
6 *	seed	<0.1	11.01 ± 0.43	79.42 ± 1.27
7 *	peel	<0.05	25.24 ± 1.10	81.2 ± 0.68
8 *	blade	<0.05	20.30 ± 1.33	63.88 ± 0.66
9 *	branch	ND	3.48 ± 0.21	63.23 ± 0.24
10 *	xylem	ND	2.39 ± 0.28	51.95 ± 0.03
11 *	bark	ND	6.85 ± 0.26	70.07 ± 0.23
12 *	root	ND	3.81 ± 0.26	46.48 ± 0.36

“*” Nos. 5–12 represent different organs of *A. sinensis* (blossom, seeds, peel, leaf, branch, xylem, bark, and root). ND: Not detected.

**Table 2:** Chemical composition analysis of different organs from *A. sinensis*.

*A. sinensis* Organ	Main Chemical Components	Molecular Formular	Molecular Weight	CAS Number	RA%	
					Volatile Component	Alcohol Extracts
blossom	Benzoic acid, 2-hydroxy-, phenylmethyl ester *n*-Hexadecanoic acid	C_14_H_12_O_3_ C_16_H_32_O_2_	228 256	118-58-1 57-10-3	16.59 --	-- 15.96
seed	10-Octadecenoic acid, methyl ester Squalene	C_19_H_36_O_2_ C_30_H_50_	296 410	13481-95-3 111-02-4	21.30 --	-- 36.66
peel	Benzoic acid, 2-hydroxy-, phenylmethyl ester Benzoic acid, ethyl ester	C_14_H_12_O_3_ C_9_H_10_O_2_	228 150	118-58-1 93-89-0	21.50 --	-- 28.28
blade	Octadecane, 3-ethyl-5-(2-ethylbutyl)-*n*-Hexadecanoic acid	C_26_H_54_ C_16_H_32_O_2_	366 256	55282-12-7 57-10-3	13.34 --	-- 8.66
branch	4-((1*E*)-3-Hydroxy-1-propenyl)-2-methoxyphenol	C_10_H_12_O_3_	180	458-35-5	--	15.07
xylem	8-Naphthol, 1-(benzyloxy)-	C_17_H_14_O_2_	250	326875-68-7	--	-- 18.18
bark	9-Octadecenoic acid, 1,2,3-propanetriyl ester	C_57_H_104_O_6_	884	537-39-3	--	6.74
root	Benzoic acid, ethyl ester	C_9_H_10_O_2_	150	93-89-0	--	20.32

RA%: Relative area in total compounds.

**Table 3:** Chemical composition analysis of agarwood from different regions.

Main Chemical Components (RA%)	Molecular Weight	Malaysia	China	Indonesia	Vietnam
Isoaromadendrene epoxide (C_15_H_24_O)	220	0.28%	1.14%	0.12%	4.50%
Agarospirol (C_15_H_26_O)	222	0.11%	0.13%	0.49%	0.66%
β-Guaiene (C_15_H_24_)	204	0.08%	0.07%	0.21%	0.21%
Benxylacatone (C_10_H_12_O)	148	0.21%	0.18%	0.30%	0.15%
2-(2phenylethyl)chromone (C_17_H_14_O_2_)	250	1.78%	1.65%	0.07%	0.03%
6-hydroxy-2-[2-(4-methoxyl-phenyl)ethyl]chromone (C_18_H_16_O_4_)		0.56%	0.49%	0.23%	0.21%
6-hydroxy-2-(2-phenylethyl)chromone (C_17_H_15_O_3_)		0.43%	0.79%	0.77%	0.17%

RA%: Relative area of total compounds.

**Table 4:** Inhibition activities of essential oils on *E.* *coli*, *B. subtilis*, and *S. aureus.*

Concentration(mg/mL)	*E. coli*		*B. subtilis*		*S. aureus*	
	Average OD (cm)	Inhibition Ratio (%)	Average OD (cm)	Inhibition Ratio (%)	Average OD (cm)	Inhibition Ratio (%)
0	2.03	0	2.05	0	2.04	0
0.2	2.15	5.58 ± 0.22	2.44	15.98 ± 0.32	2.43	16.05 ± 0.33
0.4	2.34	13.25 ± 0.43	2.72	24.63 ± 0.82	2.77	26.35 ± 0.81
0.6	2.56	20.70 ± 1.07	3.12	34.29 ± 1.30	3.36	39.29 ± 1.21
0.8	2.84	28.52 ± 1.31	3.57	42.58 ± 1.42	3.76	45.74 ± 1.24
1.0	3.09	34.30 ± 1.52	3.89	47.30 ± 1.33	4.01	49.13 ± 1.65
1.2	3.53	42.49 ± 1.88	4.39	53.30 ± 1.82	4.48	54.46 ± 1.75
1.4	3.81	46.72 ± 2.07	4.66	56.01 ± 2.31	4.82	57.68 ± 1.97
1.6	4.07	50.12 ± 2.44	4.97	58.75 ± 2.82	5.30	61.51 ± 2.65
1.8	4.40	53.86 ± 2.47	5.12	59.96 ± 2.34	5.54	63.18 ± 2.57
2.0	4.83	57.97 ± 3.44	5.23	60.80 ± 3.82	5.74	64.46 ± 3.01

**Table 5:** Climatic information of agarwood from different regions.

Origin	Geographical Location Information
China	Guangdong, China is located between latitude 20°13′~25°31′ and longitude 109°39′~117°19′; subtropical monsoon climate, annual average precipitation is 1300~2500 mm.
Malaysia	Malaysia is located between 1~7° north latitude and 97–120° east longitude, tropical rainforest climate and tropical monsoon climate, no obvious four seasons, average temperature is 26~30 °C, abundant rainfall.
Indonesia	Indonesia is located between 12° S–7° N and 96° E~140° E, tropical rainforest climate, annual average temperature is 25~27 °C, no four seasons, abundant precipitation, annual precipitation is 1600~2200 mm.
Vietnam	Vietnam is located at 8°30′~23°22′ north latitude and 102°10′~109°30′ east longitude, tropical monsoon climate, average annual rainfall is 1500~2000 mm.
